# Mechanical Study of Jian-Gan-Xiao-Zhi Decoction on Nonalcoholic Fatty Liver Disease Based on Integrated Network Pharmacology and Untargeted Metabolomics

**DOI:** 10.1155/2022/2264394

**Published:** 2022-07-08

**Authors:** Yong-Jun Cao, Han-Zhou Li, Jie Zhao, Yu-Meng Sun, Xiao-Wen Jin, Shu-Quan Lv, Jun-Yu Luo, Xi-Xing Fang, Wei-Bo Wen, Jia-Bao Liao

**Affiliations:** ^1^Department of Endocrinology, Nantong Hospital Affiliated to Nanjing University of Chinese Medicine, Nantong, China; ^2^Graduated School, Chengde Medical University, Chengde, China; ^3^Department of Endocrinology, Yunnan Provincial Hospital of Chinese Medicine, Kunming, China; ^4^Department of Endocrinology, Cangzhou Hospital of Integrated Traditional Chinese Medicine and Western Medicine of Hebei Province, Cangzhou, China; ^5^College of Traditional Chinese Medicine, Changchun University of Traditional Chinese Medicine, Changchun, China; ^6^Department of Emergency, Jiaxing Hospital of Traditional Chinese Medicine, Jiaxing, China

## Abstract

Jian-Gan-Xiao-Zhi decoction (JGXZ) has demonstrated beneﬁcial eﬀects on nonalcoholic fatty liver disease (NAFLD). However, the mechanisms by which JGXZ improve NAFLD are still unclear. *Methods.* In this study, we first used a high-fat diet (HFD) to establish a NAFLD rat model to clarify the therapeutic effect of JGXZ on NAFLD. Secondly, we used network pharmacology to predict the potential targets of JGXZ on NAFLD, and then the key targets obtained from network pharmacology were verified. Finally, we used untargeted metabolomics to study the metabolic regulatory mechanism of JGXZ. *Results.* JGXZ treatment could decrease body weight and ameliorate dyslipidemia in NAFLD model rats. H&E and oil red O staining indicated that JGXZ reduced steatosis and inﬁltration of inﬂammatory cells in the liver. In addition, network pharmacology research found that the potential targets of JGXZ on NAFLD pathway were mainly associated with improving oxidative stress, apoptosis, inflammation, lipid metabolism disorders, and insulin resistance. Further experimental verification confirmed that JGXZ could inhibit inflammation and improve oxidative stress, insulin resistance, and lipid metabolism disorders. Serum untargeted metabolomics analyses indicated that the JGXZ in the treatment of NAFLD may work through the linoleic acid metabolism, alpha-linolenic acid metabolism, tryptophan metabolism, and glycerophospholipid metabolism pathways. *Conclusions.* In conclusion, this study found that JGXZ has an ameliorative effect on NAFLD, and JGXZ alleviates the inflammatory response and oxidative stress and lipid metabolism disorders in NAFLD rats. The mechanism of action of JGXZ in the treatment of NAFLD may be related to the regulation of linoleic acid metabolism, tryptophan metabolism, alpha-linolenic acid metabolism, and glycerophospholipid metabolism.

## 1. Introduction

Nonalcoholic fatty liver disease (NAFLD) is a chronic metabolic stress liver disease, and it is one of the most common liver diseases [1]. In 2020, the global prevalence of NAFLD was as high as 25%, and it is increasing every year [2]. The widespread prevalence of NAFLD has directly led to an increase in the incidence of cirrhosis, liver cancer, and cardiovascular disease, posing a serious threat to human life and health, as well as a significant economic burden on patients, families, and society [3]. Therefore, it is essential to establish practical interventions early to interrupt or delay the progression of NAFLD.

Traditional Chinese medicine (TCM) formulas have the characteristics of having multiple ingredients, multiple targets, and multiple pathways of actions. Traditional research methods cannot illustrate the effectiveness of drugs at the molecular level, and the research results cannot effectively elucidate the complex interactions among the efficacy of prescriptions, substance components, and the biological network of the organism. Network pharmacology can explain the material basis and mechanism of action of TCM herbal formulas from the perspective of macro-systematic and complex correlation [4]. Metabolomics allows for the comprehensive analysis of endogenous small molecules in biological samples and multivariate analysis of the overall changes in endogenous metabolites after stimulation or disturbance of the organism [5]. By combining network pharmacology with metabolomics, the mechanism of action of herbal formulas can be interpreted in depth at various levels, such as molecular, pathway, and metabolism, and this combination has become very important to study the mechanism of herbal formulas. He et al. [6] discovered the mechanism of action of *Coptis chinensis* Franch., a classic formula for the treatment of type 2 diabetes mellitus, using network pharmacology combined with an untargeted metabolomics approach. Liu et al. [7] elucidated the characteristics of metabolic abnormalities in depression models through a combination of metabolomics and network pharmacology, confirming the antidepressant mechanism of Free Wanderer Powder and providing evidence to support its further clinical applications.

Jiangan Xiaozhi decoction (JGXZ) consists of *Salvia miltiorrhiza Bunge*, *Panax notoginseng*, *Curcuma zedoaria*, *Hawthorn*, *Astragalus membranaceus*, *Vatica mangachapoi Blanco*, *Radix Paeoniae Rubra*, *Curcuma longa*, *Rhizoma Alismatis*, *Dendranthema morifolium*, *Lotus leaf*, and *Glycyrrhiza uralensis Fisch* [8]. It has significant therapeutic effects on NAFLD and can effectively improve patients' clinical indicators. A previous study demonstrated that JGXZ can improve dyslipidemia and insulin resistance in an NAFLD rat model [9]. However, the multi-ingredient and multi-targeting nature of herbal medicines plays a common role in their efficacy, and it is difficult to effectively elucidate the potential mechanism of JGXZ. Therefore, in this study, we first generated a NAFLD rat model using high-fat chow to clarify the role of JGXZ in the treatment of NAFLD. Second, we used network pharmacology to predict the main targets of JGXZ for the treatment of NAFLD and validated the key targets using western blotting and other methods. Finally, untargeted metabolomics was employed to study the changes in metabolite levels in the serum of NAFLD rats treated with JGXZ. The network pharmacology results were correlated with the metabolomics results to investigate the integrated mechanism of JGXZ for the treatment of NAFLD from the molecular metabolite perspective and to investigate the mechanism of the integrative effect of JGXZ in the treatment of NAFLD.

## 2. Materials and Methods

### 2.1. Prediction of Potential Targets for JGXZ and NAFLD

We used the TCM systems pharmacology database and analysis platform (TCMSP) to screen the drug ingredients and their action targets of *Salvia miltiorrhiza Bunge*, *Panax notoginseng*, *Curcuma zedoaria*, *Hawthorn*, *Astragalus membranaceus*, *Vatica mangachapoi Blanco*, *Radix Paeoniae Rubra*, *Curcuma longa*, *Rhizoma Alismatis*, *Dendranthema morifolium*, *Lotus leaf*, and *Glycyrrhiza uralensis Fisch* in the compound formula with an oral bioavailability of ≥30% and drug-likeness of ≥0.18 [10]. As *Hawthorn* was not included in the TCMSP database, we chose the bioinformatics analysis tool for molecular mechANism of traditional Chinese medicine (BATMAN-TCM) database with a cutoff score of ≥20 [11]. This was used as the criterion to screen the active ingredients of *Hawthorn* and their targets. Then, we searched for disease-related disease targets by entering the keywords “nonalcoholic fatty liver disease” in the online Mendelian inheritance in man (OMIM) (http://omim.org/) and GeneCards (https://www.genecards.org/) database to eliminate duplicate genes and identify disease targets.

### 2.2. Target Screening and Pathway Network Construction

The obtained formula targets were intersected with the screened disease targets to obtain the potential targets of action of the compound formula against NAFLD. A network of compounds, formulas, active ingredients, target-of-action, and diseases was constructed using Cytoscape 3.8.2 software.

### 2.3. Reagents

HFD (17.7% sucrose, 17.7% fructose, 19.4% protein, and 40% fat) was purchased from Beijing Huafukang Bioscience Co., Ltd. (Beijing, China), Aspartate aminotransferase (AST; cat. no. C010-2-1), alanine aminotransferase (ALT; cat. no. C009-2-1), triglyceride (TG; cat. no. A110-1-1), and total cholesterol (TC; cat. no. A111-1-1) test kits were purchased from Nanjing Jiancheng Bioengineering Institute (Nanjing, China). The oil red O staining kit (cat. no. G1261) was obtained from Solarbio Biotechnology Co., Ltd. (Beijing, China). Superoxide dismutase (SOD; cat. no. A001-3-1), methane dicarboxylic aldehyde (MDA; cat. no. A003-1-1), and glutathione peroxidase (GSH-Px; cat. no. A005-1) assay test kits were obtained from Nanjing Jiancheng Biological Engineering Institute (Nanjing, China). Rat IL-1*β* (cat. no. E02I0010), IL-6(cat. no. E02I0006), tumor necrosis factor alpha (TNF-*α*; cat. no. E02T0008), and enzyme-linked immunosorbent assay (ELISA) kit was obtained from Shanghai BlueGene Biotech Co., Ltd. (Shanghai, China). Rabbit anti-NF*κ*B-p65 (cat. no. 51–0500), rabbit anti-phospho-NF*κ*B-p65 (cat. no. 44–711G), rabbit anti-GSK3*β* (cat. no. 44–610), rabbit anti-Fas (cat. no. MA5-14882), rabbit anti-Leptin (cat. no. PA1-051), rabbit anti-CYP2E1 (cat. no. PA5-52652), rabbit anti-cytochrome C (cat. no. 45–6100), rabbit anti- SREBP1(cat. no. MA5-11685), rabbit anti-c-JUN (cat. no. MA5-15881), rabbit anti-c-JUN (cat. no. MA5-15881), rabbit anti-JNK (cat. no. 44–690G), and primary antibodies were purchased from Invitrogen (USA).

### 2.4. Preparation of JGXZ

JGXZ contained 20 g of *Radix Paeoniae Rubra*, 15 g of *Dendranthema morifolium*, 15 g of *Salvia miltiorrhiza Bunge*, 20 g of *Hawthorn*, 6 g of *Panax notoginseng*, 15 g of *Rhizoma Alismatis*, 20 g of *Astragalus membranaceus*, 15 g of *Curcuma zedoaria*, 15 g of *Lotus leaf*, 10 g of *Vatica mangachapoi Blanco*, 12 g of *Curcuma longa*, and 6 g of *Glycyrrhiza uralensis Fisch*. These herbs were soaked in 500 mL of distilled water for 10 min and decocted for 30 min. The water extract of JGXZ was then ﬁltered and concentrated to 4 g crude herb/mL.

### 2.5. Animals

Thirty specific-pathogen free 6–8-week-old male Sprague–Dawley rats, weighing 200 ± 20 g, were provided by Beijing Huafukang Biotechnology Co., Ltd (Production License No.: SCXK (Beijing) 2019–0008). The housing environment was maintained at 25 ± 2°C, 50 ± 15% relative humidity, and a 12 h light and dark cycle, with free access to water and food. During the domestication and study periods, all the animals had free access to food and water, and were kept on a 12 h light/dark cycle (21 ± 2°C and 45 ± 10% relative humidity). This study has been approved by the Ethics Committee of Animal Medicine and Animal Care of Nanjing University of traditional Chinese medicine.

### 2.6. Generation and Grouping of the NAFLD Rat Model

After 1 week of acclimatization, the NAFLD rat model was replicated according to a previously described method [12]. Thirty rats were randomly divided into control (*n* = 10), model (*n* = 10), and JGXZ groups (*n* = 10). The control group was given normal chow, and the other groups were given high-fat chow (4% cholesterol, 10% lard, 5% sugar, 0.5% sodium cholate, 0.2% propylthiouracil, and 80.3% basal feed), both for 12 weeks. During the 12-week model generation period along with the drug intervention, the control and model groups were given 2 mL/day saline via intragastric administration, and the JGXZ group was given 16 g of JGXZ/kg of body weight [13] via intragastric administration for 12 consecutive weeks. The body weight of the rats was recorded every 2 weeks during the experiment.

### 2.7. Biochemical Indicators and Liver Index Assay

After model generation and drug administration, all the rats were fasted for 24 h and anesthetized via intraperitoneal injection of 1% phenobarbital (10 mL/kg of body weight). Blood samples were collected from the abdominal aorta and centrifuged at 4°C at 3,000 rpm for 10 min, and the serum was separated. A kit was used to determine the levels of ALT, AST, TG, and TC in the rat serum. The liver index was calculated using the following formula: liver index (%) = liver weight (g)/body weight (g) × 100%.

### 2.8. Liver Histology

Immediately after rat model generation and drug administration, liver tissues from the same part of the left lobe of the liver was removed, cleaned, fixed in 10% formalin, and dehydrated for paraffin embedding. They were cut into 5 *μ*m sections using a microtome, routinely stained with hematoxylin–eosin (H&E), transparently sealed, and observed under a light microscope to assess pathological changes in the liver.

### 2.9. Determination of Liver Oxidative Stress Parameters

One-hundred micrograms of liver tissue was added to 900 *μ*L of saline, homogenized by ultrasonication, and centrifuged at low temperature for 15 min; the supernatant was collected. The levels of SOD, GSH-Px, and MDA in the tissue homogenate were determined using the BCA kit according to the kit instructions.

### 2.10. ELISA

The levels of the cytokines TNF-*α*, IL-1*β*, and IL-6 in the serum of each group of rats were determined using ELISA according to the kit instructions.

### 2.11. Western Blotting

Western blotting was performed to detect NF-*κ*B P65 phosphorylation and the expressions of JNK1/2, SREBP1, leptin, Fas, cytochrome C, GSK3*β,* and CYP2E1 proteins in the liver tissues. Liver tissues were collected from each group, weighed at approximately 20 mg, added to 150 *μ*L of RIPA protein lysis buffer, and centrifuged. The proteins were retained after centrifugation of the homogenate. The total protein concentration was determined using the BCA protein assay kit. An equal amount of protein (10 *μ*g) was taken from each sample. The proteins were separated using SDS-PAGE electrophoresis, transferred to PVDF membranes, blocked with 5% skimmed milk for 2 h at room temperature, and incubated with rabbit anti-rat primary antibody P65 (1 : 1,000), p-P65 (1 : 1,000), JNK1/2 (1 : 2,000), SREBP1 (1 : 1,000), leptin (1 : 1,000), Fas (1 : 1,000), cytochrome C (1 : 2,000), GSK3*β* (1 : 1,000), CYP2E1 (1 : 1,000), and *β*-action (1 : 2,000) overnight at 4°C. After washing the membrane, the membrane was incubated with the secondary antibody (goat anti-rabbit IgG; 1 : 8,000) at room temperature for 2 h. After washing the membrane with Tris-buffered saline, enhanced chemiluminescence was added to develop and detect the bands with Image-Pro plus 6.0 software for quantitative analysis of the grayscale values [14].

### 2.12. Untargeted Metabolomics Assay

Untargeted metabolomics analysis was performed with 100 *μ*L serum sample using liquid chromatography-mass spectrometry. The specific sample processing procedures, chromatographic and mass spectrometric conditions, data processing, and analysis were performed as described in our previous study [15].

### 2.13. Statistical Analysis

The experimental data were analyzed using SPSS 20.0 (IBM SPSS Statistics for Windows, Version 20.0; Armonk, NY, USA). Data were expressed as mean ± standard deviation. The *t*-test was performed for comparisons between groups, and one-way analysis of variance was performed for comparisons among multiple groups. Differences were considered statistically significant at *p* < 0.05.

## 3. Results

### 3.1. Therapeutic Effect of JGXZ on NAFLD Rats

**Figure 1:**
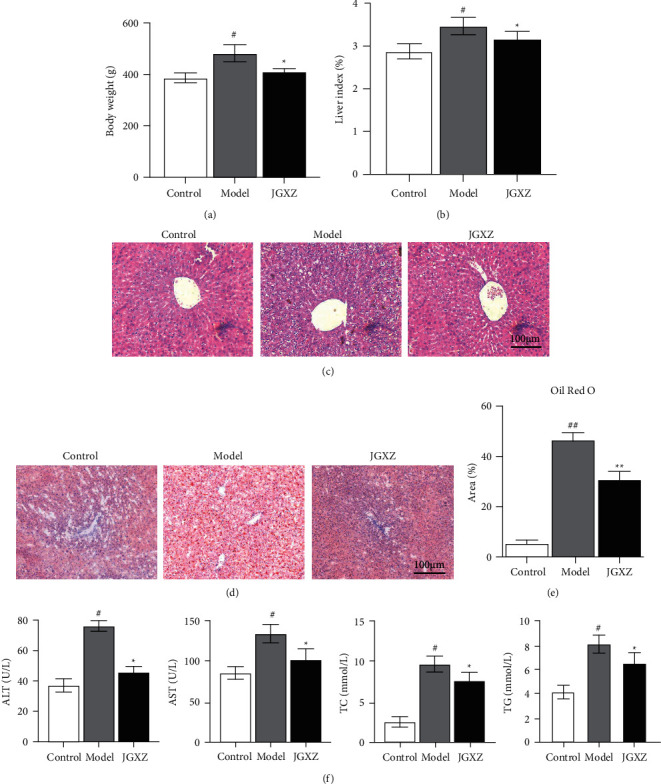
JGXZ treatment reduced body weight gain and improved liver steatosis in NAFLD model rats. (a) JGXZ treatment decreased the body weight in NAFLD model rats. (b) JGXZ treatment decreased the liver index in NAFLD model rats. (c) H&E staining indicated that JGXZ treatment ameliorated liver steatosis in NAFLD model rats (100 ×). (d, e) oil red O staining showed that after JGXZ treatment, the liver lipid content of NAFLD model rats decreased(100 ×). (f) JGXZ treatment decreased the levels of ALT, AST, TG, and TC in NAFLD model rat serum. Control, model, and JGXZ groups (*n* = 10 per group). Data are presented as the mean ± SD. #*p* < 0.05 compared to the control group. ##*p* < 0.01 compared to the control group. ^*∗*^*p* < 0.05 compared to the experimental model group.^*∗∗*^*p* < 0.01 compared to the experimental model group.

Compared with that in the control group, the body weight of the rats in the model group was significantly increased (*p* < 0.01, Figure 1(a)); compared with that in the model group, the body weight of the rats in the JGXZ group was significantly reduced (*p* < 0.01, Figure 1(a)). Compared with that in the control group, the hepatic indicators significantly increased in the model group, and compared with that in the model group, the hepatic indicators in the JGXZ group decreased (*p* < 0.01, Figure 1(b)). As shown in Figure 1(c), the liver of the rats in the control group was structurally intact, with hepatocytes arranged neatly in a radial pattern; the morphology of hepatocytes around the portal area and central vein was clear and intact, and the nuclei were uniform in size and shape and located in the center of the hepatocytes. The liver of rats in the model group showed a significant extent of steatosis in the hepatocytes, the hepatocytes were distinctly swollen and rounded, the nuclei were squeezed to one side, the cytoplasm was filled with a large number of fat vacuoles, the lipid droplets were of different sizes and even fused into large droplets, and inflammatory cell infiltration was observed. Treatment in the JGXZ group significantly alleviated steatosis and inflammatory cell infiltration caused by the high-fat chow diet (Figure 1(c)). The results of oil red O staining (Figures 1(d) and 1(e)) indicated that no red lipid droplets appeared in hepatocytes in the control group, red lipid droplets appeared in hepatocytes in the model group, and the number of red lipid droplets in hepatocytes in the JGXZ group were significantly reduced. In addition, the activity of serum AST and ALT and the levels of TC and TG were significantly increased in the model group compared with those in the control group (all *p* < 0.01, Figure 1(f)). Compared with that in the model group, JGXZ significantly reduced the levels of AST, ALT, TC, and TG (all *p* < 0.01, Figure 1(f)).

### 3.2. Network Pharmacology Results

#### 3.2.1. Active Ingredients and Targets of Action in the Compound Formula

**Figure 2:**
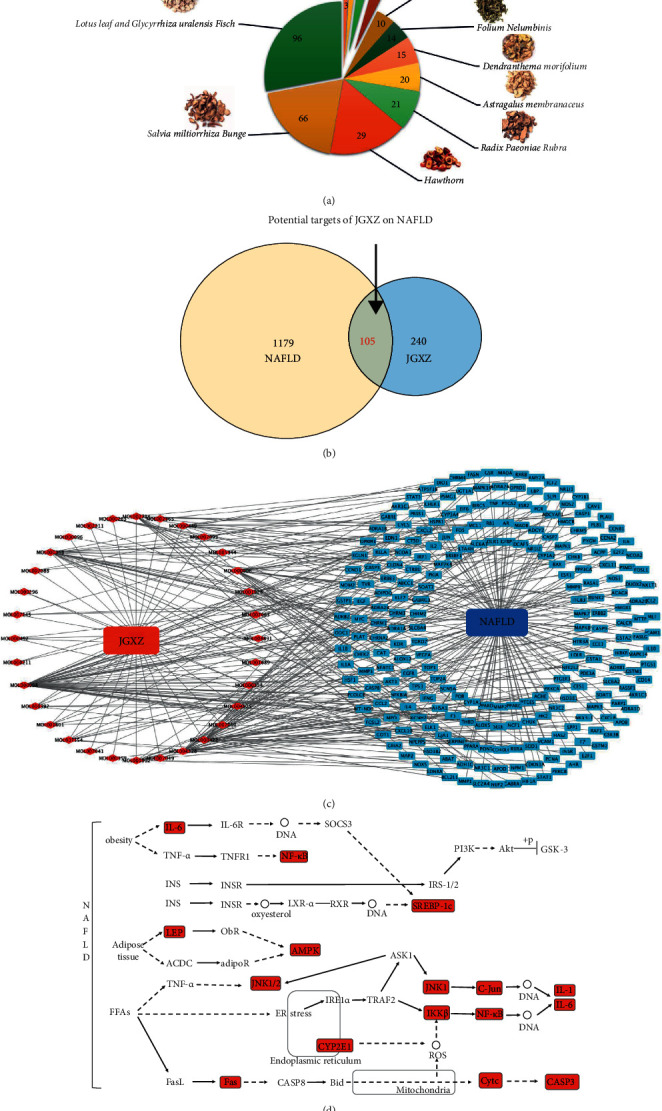
(a) The number of active ingredients of each herb in JGXZ obtained from TCMSP (OB ≥ 30%, DL ≥ 0.18) and BATMAN-TCM(score cutoff ≥20). (b) Veen diagram of compound targets of JGXZ and NAFLD-related targets. (c) Drug-component-disease-target network (Red diamond nodes represented the active ingredient in JGXZ and blue rectangle nodes represented the potential targets). (d) Overview of potential targets of JGXZ on NAFLD pathway (map04932) based on KEGG analysis (the potential targets are shown in red).

Based on the TCMSP and BATMAN-TCM databases, there were 343 compounds in 12 Chinese herbs of the JGXZ formula (Figure 2(a)), including 66 in *Salvia miltiorrhiza Bunge*, 8 in *Panax notoginseng*, 4 in *Curcuma zedoaria*, 21 in *Astragalus membranaceus*, 14 in *Vatica mangachapoi Blanco*, 29 in *Radix Paeoniae Rubra*, 3 in *Curcuma longa*, 10 in *Rhizoma Alismatis*, 20 in *Dendranthema morifolium*, 15 in *Lotus leaf*, 96 in *Glycyrrhiza uralensis Fisch*, and 57 in *Hawthorn*. After removing the duplicate ingredients, a total of 32 compounds were obtained. The detailed results are shown in Supplementary Table S1. After pairwise comparison of the TCMSP and BATMAN-TCM databases, a total of 1186 active targets of JGXZ compounds were obtained, and 240 active targets of JGXZ compounds were obtained by uniport comparison and de-duplication. In addition, 1179 NAFLD targets were obtained from the OMIM and GeneCards databases, where the compound formula and disease were further intersected to obtain component targets (Figure 2(b)), which screened out a total of 156 active ingredients with potential anti-NAFLD activity in the formula, 176 targets in the compound formula, and 1,392 targets of disease, and 66 potential targets of anti-NAFLD activity in the formula were obtained after intersecting the two.

### 3.3. Network Analysis

Cytoscape 3.8.2 was used to construct a compound–formula–active-ingredient–target-of-action–disease network, and the core nodes were screened based on the network topological features (Figure 2(c)). We analyzed these targets against “NAFLD (map04932)” in the Kyoto Encyclopedia of Genes and Genomes database (Figure 2(d)) and found that JGXZ targets in the NAFLD pathway mainly included P65, JNK1/2, C-JUN, SREBP1, leptin, GSK3*β*, CYP2E1, Fas, and cytochrome C. These targets are mainly associated with oxidative stress, apoptosis, inflammation, lipid metabolism disorders, insulin resistance, and insulin resistance. We selected oxidative stress and apoptosis-related proteins Fas and cytochrome C, inflammation-related proteins P65 and JNK1/2, lipid metabolism disorder-related proteins SREBP1 and leptin, and insulin resistance-related proteins GSK3*β* and CYP2E1 for subsequent experimental validation.

### 3.4. Effects of JGXZ on Oxidative Stress and Apoptosis in the NAFLD Rat Model

**Figure 3:**
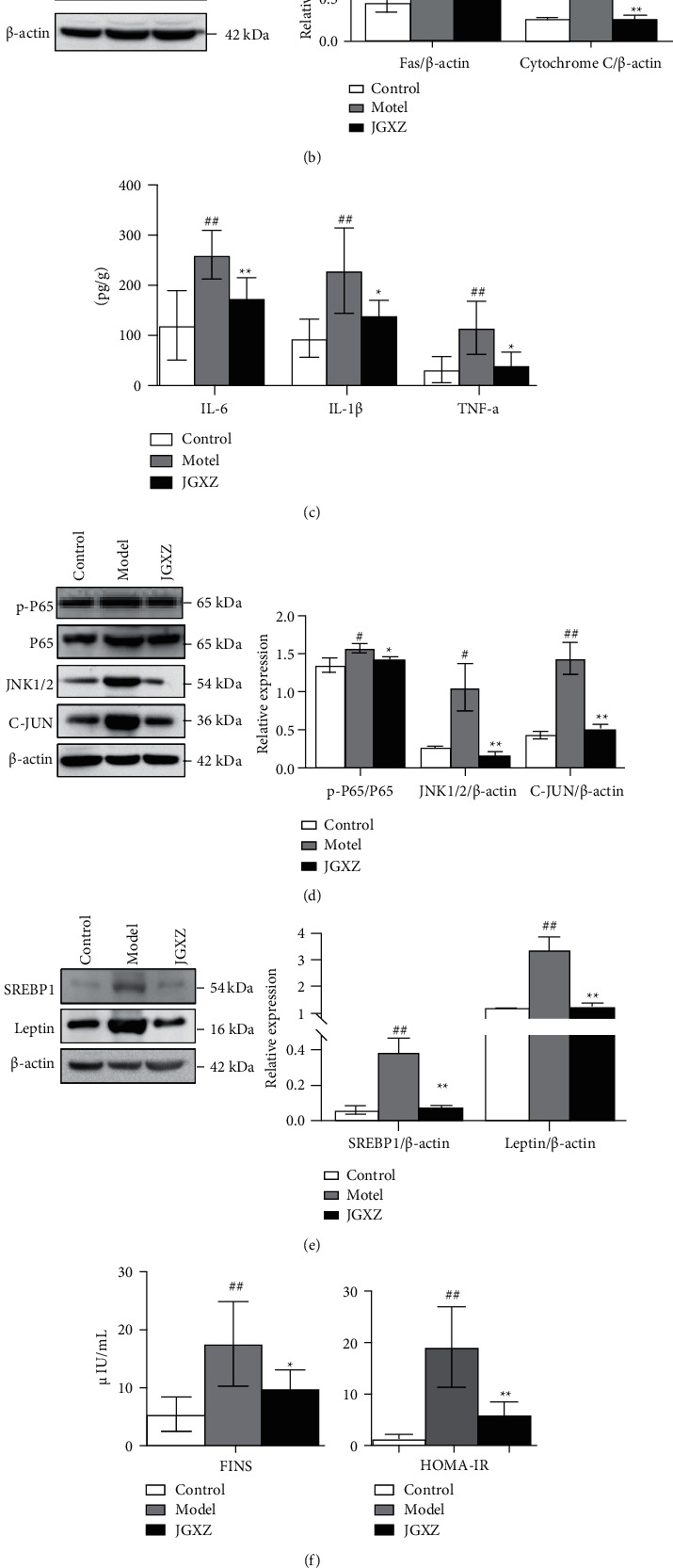
Experimental validation of network pharmacology analysis (a) JGXZ treatment increased the SOD and GSH-Px activities, and reduced the MDA level in the liver tissue homogenate. (b) JGXZ downregulated the protein expressions of Fas and Cytochrome C expression in the liver tissue. (c) JGXZ treatment decreased the levels of pro-inflammatory cytokines in the liver tissues. (d) JGXZ downregulated the protein expressions of NF-*κ*B p65 phosphorylation, JNK1/2, and C-JUN in the liver tissue. (e) JGXZ downregulated the protein expressions of SERBP1 and Leptin in the liver tissue. (f) JGXZ treatment reduced the FINS level and HOMA-IR in NAFLD model rats. (g) JGXZ downregulated the protein expressions of GSK3*β* and CYP2E1 in the liver tissue.

We evaluated the effects of JGXZ on oxidative stress and apoptosis in NAFLD rats by measuring the activities of SOD and GSH-Px and levels of MDA, Fas, and cytochrome C in the liver tissues of the rats in each group. Compared with that in the control group, the SOD and GSH-Px activities in the liver of rats in the model group were significantly decreased (*p* < 0.01), and MDA levels were significantly increased (*p* < 0.01). JGXZ treatment significantly increased the SOD and GSH-Px activities and decreased the MDA level in the liver of NAFLD rats (*p* < 0.01 and *p* < 0.05, Figure 3(a)). Western blotting results showed that the levels of Fas and cytochrome C were elevated in the liver of rats in the model group compared with those in the control group (*p* < 0.01, Figure 3(b)) and were significantly decreased after JGXZ treatment compared with those in the model group (*p* < 0.01, Figure 3(b)). These results suggest that JGXZ inhibits oxidative stress and apoptosis in NAFLD by downregulating the protein expressions of Fas and cytochrome C in the liver tissues.

### 3.5. Effects of JGXZ on Inflammation in the NAFLD Rat Model

To investigate the effects of JGXZ on the inflammatory response in the liver tissues of NAFLD rats, we performed ELISA to determine the levels of the proinflammatory factors IL-6, IL-1*β* and TNF-*α* in the liver tissues. The levels of IL-6, IL-1*β* and TNF-*α* were significantly increased in the liver tissues of rats in the model group compared with those in the control group (*p* < 0.01), and JGXZ treatment significantly reduced the levels of IL-6, IL-1*β*, and TNF-*α* in the liver tissues of NAFLD rats compared with that in the model group (*p* < 0.01 and *p* < 0.05, Figure 3(c)). We performed western blotting to determine the levels of inflammation-related protein NF-*κ*B p65 phosphorylation, JNK1/2, and C-JUN levels in the liver tissues. NF-*κ*B p65 phosphorylation, JNK1/2, and C-JUN levels were elevated in the liver tissues of rats in the model group compared with that in the control group (*p* < 0.01, Figure 3(d)), which were significantly decreased after JGXZ treatment compared with that in the model group (*p* < 0.01, Figure 3(d)). These results indicate that JGXZ may alleviate the inflammatory response to NAFLD by downregulating NF-*κ*B p65 phosphorylation, JNK1/2, and C-JUN levels in the liver tissues and reducing inflammatory cytokine levels.

### 3.6. Effects of JGXZ on Lipid Metabolism in the NAFLD Rat Model

Western blotting was performed to determine the levels of SREBP1 and leptin in the liver tissues. The results showed that the levels of related proteins were increased in the model group compared with those in the control group (*p* < 0.01, Figure 3(e)), and JGXZ treatment significantly reduced the levels of related proteins compared with those in the model group (*p* < 0.01, Figure 3(e)). These results suggest that JGXZ alleviates NAFLD insulin resistance by inhibiting SREBP1 and leptin activity.

### 3.7. Effects of JGXZ on Insulin Resistance in the NAFLD Rat Model

We calculated the homeostatic model assessment for the insulin resistance (HOMA-IR) index. The results showed that the fasting insulin (FINS) level and HOMA-IR index were significantly increased in the model group rats compared with those in the control group (*p* < 0.05), and JGXZ treatment significantly reduced the levels of FINS and HOMA-IR index in the model group (*p* < 0.05 and *p* < 0.01, respectively, Figure 3(f)). We performed western blotting to determine the levels of GSK3*β* and CYP2E1 in the liver tissues. The results showed that the model group had elevated levels of related proteins compared with those in the control group. Compared with those in the model group, the levels of related proteins were significantly reduced after JGXZ treatment (*p* < 0.01 and *p* < 0.05, Figure 3(g)). These results suggest that JGXZ alleviates NAFLD insulin resistance by inhibiting GSK3*β* and CYP2E1 activity.

### 3.8. Effects of JGXZ on Serum Metabolite Levels in the NAFLD Rat Model

**Figure 4:**
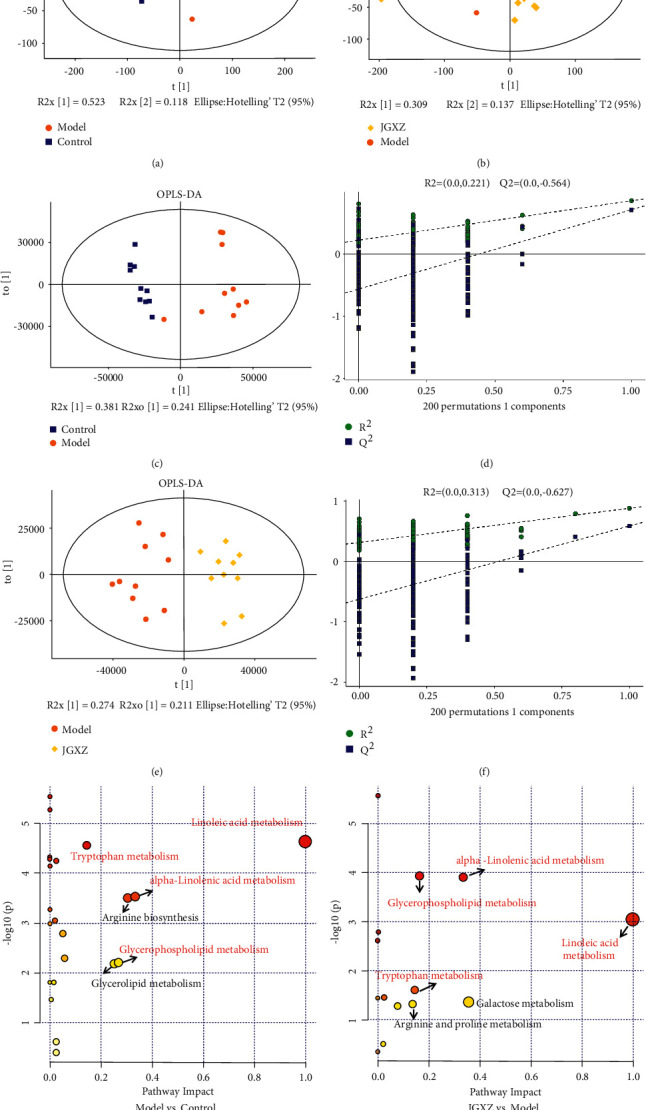
(a) PCA scores of control group and model group. (b) PCA scores of JGXZ group and model group. (c, d) OPLS-DA score and load coefficient diagram of control group and model group. (e, f) OPLS-DA score and load coefficient diagram of model group and JGXZ group. (g) Pathway analysis diagram of control group and model group. (h) Pathway analysis diagram of model group and JGXZ group. (a) linoleic acid metabolism; (b) tryptophan metabolism; (c) alpha-linolenic acid metabolism; (d) arginine biosynthesis; (e) glycerophospholipid metabolism; (f) glycerolipid metabolism; (g) galactose metabolism; (h) arginine and proline metabolism. The common pathways have been marked in red.

Principle component analysis (PCA) plots showed that the control group was well distinguished from the model group and that the model group was well distinguished from the JGXZ group (Figures 4(a), 4(b)). An orthogonal partial least squares discriminant analysis (OPLS-DA) model was used for differential metabolite identification, and the explanatory rate (*R*^2^) and predictive power (*Q*^2^) of the model were evaluated by applying seven-round cross validation and 200 repetitions of RPT under the established OPLS-DA model. *R*^2^ was 0.0, R0.221 and *Q*^2^ was 0.0, −0.564 between the control and model groups(Figures 4(c) and 4(d)), and *R*^2^ was 0.0, 0.313 and *Q*^2^ was 0.0, −0.627 between the model and JGXZ groups (Figures 4(e), 4(f)).

Furthermore, differential metabolites (*p* < 0.05 and VIP >1) were identified based on our previous study [16], as shown in Table 1. Compared with the control group, the serum levels of L-proline, L-lysine, citrulline, L-tryptophan, L-isoleucine, L-valine, L-arginine, sphingosine-1-phosphate, L-leucine, glycocholic acid, uric acid, stearic acid, glyceryl phosphoryl ethanolamine, palmitic acid, TG (18 : 0/20 : 4 (5Z,8Z,11Z,14Z)/20 : 4 (5Z,8Z,11Z,14Z)), phosphatidylethanolamine, and glycerol. The contents of 12(R)-HETE and D-galactose were significantly increased; while the contents of acetylcholine, linoleic acid, eicosapentaenoic acid, alpha-linolenic acid, gluconic acid, choline, phosphatidylcholine, and L-threonine were significantly decreased. The contents of L-threonine, acetylcholine, linoleic acid, eicosapentaenoic acid, alpha-linolenic acid, gluconic acid, and choline in the rat serum were significantly increased, L-proline, L-lysine, citrulline, L-tryptophan, L-Iioleucine, *L* -valine, L-arginine, sphingosine-1-phosphate, L-leucine, glycocholic acid, uric acid, stearic acid, glyceryl phosphoryl ethanolamine, palmitic acid, TG(18 : 0/20 : 4(5Z,8Z,11Z,14Z)/20 : 4(5Z, 8Z, 11Z, 14Z)), phosphatidylethanolamine, glycerol, and 12 (R)-HETE, D-galactose were significantly decreased (Table 1).

Metabolite pathway enrichment analysis of differential metabolites in NAFLD model rats was conducted using Metabo Analyst website (Figures 4(g) and 4(h)). Differential metabolic pathways (Pathway impact >0.10, *p* < 0.05) were identified based on our previous study [16]and the differential metabolic pathways between the control group and the model group included linoleic acid, tryptophan metabolism, alpha-linolenic acid metabolism, arginine biosynthesis, glycerophospholipid metabolism, glycerolipid metabolism. The differential metabolic pathways between the model group and the JGXZ group include linoleic acid metabolism, tryptophan metabolism, alpha-linolenic acid metabolism, glycerophospholipid metabolism, galactose metabolism, arginine and proline metabolism. Among them, the pathways of linoleic acid metabolism, alpha-linolenic acid metabolism, tryptophan metabolism, and glycerophospholipid metabolism are the pathways shared by normal and model, model and JGXZ group. These pathways were selected as the metabolic pathways of JGXZ interfering with NAFLD and discussed.

## 4. Discussion

In this study, a high-fat chow diet was used to establish an NAFLD rat model. The TG, TC, ALT, and AST levels were significantly elevated in the liver tissues of rats in the model group. Pathological examination also showed significant steatosis and cellular damage in hepatocytes in the model group, suggesting the successful generation of the NAFLD model, in agreement with previous studies [15]. The JGXZ group had lower TG, TC, ALT, and AST levels after treatment and improved liver pathological alterations, which suggests that JGXZ has a therapeutic impact on NAFLD, which is consistent with our previous research findings.

Oxidative stress and apoptosis are important factors during the NAFLD development [17]. We found that JGXZ was able to increase the SOD and GSH-Px activities and decrease the MDA level in NAFLD rats, which suggested that JGXZ could attenuate oxidative stress and apoptotic responses in NAFLD rats. MDA is a product of lipid peroxidation caused by free radicals or reactive oxygen species (ROS) in cells under oxidative stress, and MDA levels indirectly reflect the degree of oxidative damage in cells [18]. SOD and GSH-Px are antioxidant enzymes that respond to antioxidant capacity [19]. SOD acts as an intracellular oxygen radical scavenger, catalyzing the formation of O_2_^−^ from O_2_ and H_2_O_2_, thereby protecting the organism from superoxide anions. GSH-Px catalyzes the conversion of reduced GSH to oxidized glutathione, protecting cells from disruption and damage caused by peroxide. The network pharmacology results identified Fas and cytochrome C, which are important proteins related to oxidative stress and apoptosis. Our results revealed that the levels of Fas and cytochrome C in NAFLD rats decreased by varying extents after JGXZ treatment. Fas acts as a protein receptor molecule on the cell surface. FasL is a ligand for Fas and can bind with Fas to initiate apoptotic signaling after stimulated by lipotoxicity factors [20]. Cytochrome C is an important protein that reflects cellular oxidative stress and apoptosis. Upon release into the cytoplasm, cytochrome C binds to apoptosis protease-activating factor-1, activates caspase-9 and triggers apoptosis [21].

The inflammatory response is an important pathological process in NAFLD, and the imbalance of inflammatory factors in NAFLD is an important pathological basis for hepatocyte damage during the progression of the disease [22]. Proinflammatory factors such as IL-6, IL-1*β*, and TNF-*α* exacerbate NAFLD hepatocyte damage in response to increased inflammation [23]. We found that JGXZ reduced the levels of IL-6, IL-1*β*, and TNF-*α* in NAFLD model rats. The results of network pharmacology revealed that the inflammation-related proteins P65 and JNK1/2 may be the targets of action of JGXZ. Further experimental validation revealed that JGXZ significantly reduced NF-*κ*B p65 phosphorylation and JNK1/2 levels in the liver. P65 is an important subunit involved in NF-*κ*B activation and is normally in a nonactivated state. Upon stimulation, NF-*κ*B p65 phosphorylation degrades I*κ*B, leading to the nuclear translocation of NF-*κ*B, which in turn stimulates the release of inflammatory cytokines and exacerbates inflammatory damage. Jun N-terminal kinases (JNK) are members of the p38 mitogen-activated protein kinase family [9]. Phosphorylation of JNK regulates the activity of downstream products, which affects the activation of inflammatory cells and the release of inflammatory cytokines, e.g., TNF-*α*, IL-1*β*, and IL-6 [24]. A study found that inhibition of JNK activation improved the inflammatory response in a mouse model of inflammation and found that the release of inflammatory markers, such as IL-1*β*, TNF-*α*, and IL-6, was significantly reduced [25].

Disorders of lipid metabolism are one of the causes of the NAFLD development [26]. The results of network pharmacology revealed that SREBP1 and leptin, important regulatory proteins of lipid metabolism, may be the targets of action of the JGXZ formula, and further experimental validation confirmed that JGXZ can decrease SREBP1 and leptin levels. SREBP1 is an important transcriptional regulator that regulates lipid synthesis and exists as an inactive precursor in the endoplasmic reticulum after synthesis [27]. When the insulin signaling pathway is activated, SREBP1 can be transported from the endoplasmic reticulum to the golgi apparatus, where it is processed and cleaved by proteases. After maturation, N-SREBP1 enters the nucleus and induces the expression of genes related to lipid synthesis. Leptin exerts its biological effects through the mediation of specific receptors (Ob-R), which are class I cytokine receptors. Studies have shown that at least five subtypes of Ob-R (Ob-Ra, Ob-Rb, Ob-Rc, Ob-Rd, and Ob-Rf) are present in humans. In lipid metabolism, leptin regulates lipid metabolism by binding to Ob-Rb, activating Janus kinase signaling and signal transducer and activator of transcription pathways in both directions and affecting the secretion of various neuroendocrine hormones, such as neuropeptide [28].

Insulin resistance is closely related to the development of NAFLD. The imbalance between energy intake and expenditure leads to insulin resistance in liver tissue, causing fat accumulation in the liver and exacerbating NAFLD [29]. The results of network pharmacology showed that the regulation of insulin-related proteins GSK3*β* and CYP2E1 was the target of action of JGXZ. JGXZ significantly reduced the protein levels of GSK3*β* and CYP2E1. GSK-3 is a protein kinase that includes two isoforms, GSK-3*α*, and GSK-3*β*. GSK-3*β* is a key enzyme in hepatic glucose metabolism, and under physiological conditions, insulin functions to promote glycogen synthesis by inactivating GSK-3*β* through phosphorylation in the insulin signaling pathway. In contrast, in the presence of insulin resistance in the body, insulin sensitivity decreases and dephosphorylated GSK-3*β* increases, resulting in reduced glycogen synthesis and increased blood glucose [30]. CYP2E1 is a key enzyme in liver diseases, and excessive activation of CYP2E1 increases the formation of “electron leakage” in mitochondria, leading to massive production of ROS, which induces oxidative stress injury when the production of ROS exceeds the capacity of the cellular antioxidant defense system [31]. Then, via oxidative alteration of important insulin downstream signaling pathways, it inhibits insulin signaling function and contributes to the development of insulin resistance [32].

### 4.1. Metabolomics Analysis

Serum untargeted metabolomics was employed to investigate the effects of JGXZ on metabolites in the serum of NAFLD rats. PCA and OPLS-DA analyses indicated that the serum metabolism of NAFLD mice showed significant changes and that the serum metabolism level of NAFLD mice could be significantly affected after JGXZ treatment. Further differential metabolite analysis showed that JGXZ could affect the levels of glyceryl phosphoryl ethanolamine, alpha-linolenic acid, phosphatidylethanolamine, choline, and 27 other metabolites. Metabolic pathway analysis of the differential metabolites using MetaboAnalyst showed that the linoleic acid metabolism, alpha-linolenic acid metabolism, tryptophan metabolism, and glycerophospholipid metabolism pathways changed in the control, model, and JGXZ groups, which suggests that the effects of the JGXZ in the treatment of NAFLD may work through these pathways.

### 4.2. Linoleic Acid Metabolism

JGXZ elevated the serum level of linoleic acid in NAFLD rats. Linoleic acid is the most abundant polyunsaturated fatty acid. As an essential nutrient, linoleic acid is mainly present in vegetable oils and has a variety of biological activities, which are important for metabolic disorders. G-protein coupled receptor120 (GPR120) is a specific receptor for long-chain polyunsaturated fatty acids in the body. Linoleic acid activates two downstream signaling pathways, Gaq and *β*-arrestin-2, by binding to GPR120, which plays a role in regulating lipid metabolism, increasing insulin sensitivity, and has anti-inflammatory effects [33]. Studies have shown that linoleic acid modifies the activity of lipoprotein lipase to regulate lipid metabolism homeostasis. Lipoprotein lipase degrades TG to glycerol and free fatty acids, which is a key enzyme in lipid metabolism [34]. Its main derivative, conjugated linoleic acid, improves high-fat diet-induced hepatic lipid accumulation by increasing hepatic TAG secretion rate (TAG-SR) and *β*-oxidation as well as reducing hepatic fatty acid uptake [35]. In a mouse model of lipopolysaccharide (LPS) induced acute inflammation, regulation of linoleic acid metabolism by promoting FADS1/FASDS2/ELOV2 expression and decreasing PLA2 expression ameliorated LPS-induced acute inflammation and multiorgan injury [36]. The regulation of JGXZ in lipid metabolism disorders and its anti-inflammatory effects may be related to linoleic acid metabolism.

### 4.3. Alpha-Linolenic Acid Metabolism

JGXZ elevated the serum alpha-linolenic acid level in NAFLD rats. Alpha-linolenic acid is a plant-derived *n*-3 polyunsaturated fatty acid that has important roles in reducing TG levels, increasing fatty acid catabolism, and inhibiting inflammation. Alpha-linolenic acid increases mitochondrial activity and reduces intracellular oxidative stress. Alpha-linolenic acid ester of plant sterols (PS-ALA) is a derivative of alpha-linolenic acid that improves mitochondrial function and oxidative stress and exerts a protective effect against NAFLD [37]. *In vivo* experiments showed that alpha-linolenic acid was effective in alleviating endoplasmic reticulum stress and improving mitochondrial function, thereby improving NAFLD [38]. The alleviation of oxidative stress by the JGXZ formula may be related to alpha-linolenic acid metabolism.

### 4.4. Tryptophan Metabolism

JGXZ significantly reduced the serum L-tryptophan level in NAFLD rats. Tryptophan is one of the 20 common amino acids and an essential amino acid that is metabolized in humans mainly through the kynurenine metabolic pathway [39]. Tryptophan and its metabolites were found to have significant anti-inflammatory and antioxidant effects [40]. In animal liver, tryptophan is involved in protein synthesis, especially when there is an acute inflammatory response in the liver. Increased protein synthesis leads to an increased demand for tryptophan, which leads to a decrease in plasma levels of tryptophan. The alleviation of inflammation and oxidative stress may be potentially associated with tryptophan metabolism.

### 4.5. Glycerophospholipid Metabolism

JGXZ elevated the serum acetylcholine (ACh) levels in glycerophospholipid metabolism in NAFLD rats. Glycerophospholipids are the most abundant phospholipids in the organism and are the main components of biological membranes, participating in various biological processes, such as membrane fusion, endocytosis, and membrane transport [41]. ACh is the transmitter of the cholinergic nervous system, and the ACh receptor binds to ACh. ACh receptors are categorized into muscarinic ACh receptors (M receptors) and nicotinic ACh receptors (N receptors) according to their receptor structure and response to drug treatment. ACh has been suggested to play an important role in regulating the inflammatory response. The inflammatory stimulus signal is transmitted to the brain via the vagus nerve. After sophisticated integration by the central nervous system, ACh is released from the efferent vagal nerve terminals, and cholinergic receptors bind to and activate the transmitter, further reducing the production and release of various inflammatory cytokines and regulating the local and systemic inflammatory response of the body. Thus, JGXZ can regulate ACh levels and improve the inflammatory response in NAFLD through the glycerophospholipid metabolism pathway.

In conclusion, this study found that JGXZ has an ameliorative effect on NAFLD, and JGXZ alleviates the inflammatory response and oxidative stress and lipid metabolism disorders in NAFLD rats. The mechanism of action of JGXZ in the treatment of NAFLD may be related to the regulation of linoleic acid metabolism, tryptophan metabolism, alpha-linolenic acid metabolism, and glycerophospholipid metabolism.

Control, model, and JGXZ (*n* = 10 per group) groups. Data are presented as the mean ± SD. ^#^*p* < 0.05 as compared to the control group; ##*p* < 0.01 as compared to the control group; ^*∗*^*p* < 0.05 as compared to the model group; ^*∗∗*^*p* < 0.01 as compared to the model group.

## 5. Conclusion

This study found that JGXZ has an ameliorative effect on NAFLD, and JGXZ alleviates the inflammatory response and oxidative stress and lipid metabolism disorders in NAFLD rats. The mechanism of action of JGXZ in the treatment of NAFLD may be related to the regulation of linoleic acid metabolism, tryptophan metabolism, alpha-linolenic acid metabolism, and glycerophospholipid metabolism.

## Figures and Tables

**Table 1:** The differential metabolites in the serum after JGXZ treatment.

m/z	Rt (min)	Formula	Metabolites	VIP		FC		Trend		Pathway
				M vs. C	J vs. M	M vs. C	J vs. M	M vs. C	J vs. M	
116.07	1.06	C_5_H_9_NO_2_	L-Proline	2.43	1.07	4.47	0.49	↑^##^	↓^*∗∗*^	*h*
120.07	0.92	C_4_H_9_NO_3_	L-Threonine	1.18	1.29	0.27	2.30	↓	↑^*∗∗*^	—
147.11	0.76	C_6_H_14_N_2_O_2_	L-Lysine	1.38	0.45	2.14	0.99	↑^##^	↓	—
146.12	1.05	C_7_H_15_NO_2_	Acetylcholine	1.18	0.43	0.68	1.07	↓^#^	↑	*e*
198.08	0.96	C_6_H_13_N_3_O_3_	Citrulline	1.47	0.89	3.13	0.77	↑^##^	↓	*d*
203.08	5.77	C_11_H_12_N_2_O_2_	L-Tryptophan	1.52	2.02	2.62	0.59	↑^##^	↓^*∗∗*^	*b*
132.10	2.69	C_6_H_13_NO_2_	L-Isoleucine	2.94	0.55	2.19	0.86	↑^##^	↓	—
118.09	1.43	C_5_H_11_NO_2_	L-Valine	2.79	0.66	2.72	0.85	↑^##^	↓	—
281.25	0.71	C_18_H_32_O_2_	Linoleic acid	1.28	1.02	0.76	2.05	↓^##^	↑^*∗∗*^	*a*
175.12	0.88	C_6_H_14_N_4_O_2_	L-Arginine	2.77	1.45	1.87	0.48	↑^#^	↓^*∗∗*^	*d*, *h*
380.25	9.54	C_18_H_38_NO_5_P	Sphingosine-1-phosphate	2.17	1.60	1.97	0.51	↑^##^	↓^*∗∗*^	—
132.10	2.92	C_6_H_13_NO_2_	L-Leucine	3.89	1.74	1.77	0.68	↑^#^	↓	—
301.22	10.42	C_20_H_30_O_2_	Eicosapentaenoic acid	1.06	1.79	0.31	2.86	↓^##^	↑^*∗∗*^	—
464.30	8.80	C_26_H_43_NO_6_	Glycocholic acid	1.73	1.78	2.31	0.25	↑^#^	↓^*∗∗*^	—
169.03	2.30	C_5_H_4_N_4_O_3_	Uric acid	2.37	0.09	3.31	0.90	↑^##^	↓	—
283.26	14.23	C_18_H_36_O_2_	Stearic acid	2.30	2.07	1.72	0.61	↑^#^	↓^*∗*^	—
216.06	6.60	C_5_H_14_NO_6_P	Glyceryl phosphoryl ethanolamine	1.22	1.96	2.58	0.49	↑^#^	↓^*∗*^	*e*
255.23	11.44	C_16_H_32_O_2_	Palmitic acid	2.09	0.61	2.83	0.84	↑^##^	↓	—
277.22	10.50	C_18_H_30_O_2_	Alpha-linolenic acid	1.60	1.98	0.44	2.11	↓^##^	↑^*∗∗*^	*c*
241.06	9.83	C_6_H_12_O_7_	Gluconic acid	1.23	0.43	0.63	1.08	↓^##^	↑	—
948.80	0.84	C_61_H_102_O_6_	TG(18 : 0/20 : 4(5Z,8Z,11Z,14Z)/20 : 4(5Z,8Z,11Z,14Z))	1.96	0.59	2.24	0.95	↑^##^	↓	*f*
104.11	10.17	C_5_H_13_NO	Choline	2.19	1.84	0.70	1.76	↓^#^	↑^*∗∗*^	*e*
766.48	10.58	C_45_H_70_NO_8_P	Phosphatidylcholine	1.33	0.56	0.36	1.02	↓^##^	↑	*e*
738.55	9.33	C_42_H_80_NO_8_P	Phosphatidylethanolamine	1.13	1.08	2.74	0.46	↑^##^	↓^*∗∗*^	*a*, *c*, *e*
115.04	1.06	C_3_H_8_O_3_	Glycerol	0.25	0.04	2.79	0.98	↑^##^	↓	*f*
319.23	9.43	C_20_H_32_O_3_	12(R)-HETE	0.68	1.90	2.44	0.56	↑^##^	↓^*∗∗*^	—
181.07	10.14	C_6_H_12_O_6_	D-Galactose	0.16	1.14	1.30	0.71	↑^##^	↓^*∗∗*^	*g*

Control, model, and JGXZ (*n* =  10 per group) groups. ^#^*p* < 0.05 as compared to the control group; ^##^*p*  < 0.01 as compared to the control group; ^*∗*^*p* < 0.05 as compared to the model group; ^*∗∗*^*p* < 0.01 as compared to the model group; ↑: content increased; ↓: content decreased; vs.: versus; C: control group; M:model group; J:JGXZ group; Rt: retention time; VIP: variable importance of projection; FC: fold change *a*: linoleic acid metabolism; *b*: tryptophan metabolism; *c*: alpha-linolenic acid metabolism; *d*: arginine biosynthesis; *e*: glycerophospholipid metabolism; *f*: glycerolipid metabolism; *g*: galactose metabolism; *h*: arginine and proline metabolism.

## Data Availability

The raw data supporting the conclusions of this article will be made available by the corresponding authors, without undue reservation.

## Supplementary Materials

Supplementary Materials Information of active ingredients in JGXZ (Table S1).
